# Development and Testing of a Rocket-Based Sensor for Atmospheric Sensing Using an Unmanned Aerial System

**DOI:** 10.3390/s24061768

**Published:** 2024-03-09

**Authors:** Ryan Thalman

**Affiliations:** Department of Chemistry, Snow College, Richfield, UT 84701, USA; ryan.thalman@snow.edu

**Keywords:** sonde measurements, rocketsonde, UAS, drone, atmospheric measurements, atmospheric chemistry

## Abstract

Measurements of the vertical structure of the lower atmosphere are important to the understanding of air quality. Unmanned Aerial Systems (UASs, drones) can provide low cost, repeatable measurements of the temperature, pressure, and relative humidity. A set of inexpensive sensors controlled with an Arduino microprocessor board were tested on a UAS against a meteorology grade sensor. Two modes of operation for sampling were tested: a forward moving sampler and a vertical ascent sampler. A small particle sensor (Sensiron SPS30) was integrated and was capable of retrieving vertical aerosol distributions during an inversion event. The thermocouple-based temperature probe and the relative humidity measurement on the Bosch BME280 sensor correlated well with the meteorological sensor. The temperature and relative humidity sensors were then deployed on a rocket sounding platform. The rocket sounding system performed well up to a height of 400 m. The inexpensive sensors were found to perform adequately for low-cost development and uses in education and research.

## 1. Introduction

The horizontal and vertical distribution of pollutants and their evolution in time impact the understanding and risks associated with air quality. Air quality impacts human health as well as visibility [1,2]. Meteorology often impacts the severity of air pollution exposure due to stagnant air masses (inversions) or low horizontal or vertical mixing [3]. Understanding the boundary layer height and other meteorological variables is valuable in understanding the evolution of the tropospheric boundary layer [4]. Trace gas and aerosol concentrations may also vary with altitude depending on the extent or lack of vertical mixing. Typical boundary layer evolution includes a shallow limited boundary layer at night, with the boundary layer expanding as the sun rises and heats air near the surface causing the warm air to rise, mixing the air vertically as it does so [5,6]. The cooling warm air parcels rising from the surface are described by the dry adiabatic lapse rate (−0.0065 Km^−1^ or roughly a drop of 1 °C per 100 m of altitude gained).

A variety of methods exist for sampling meteorological as well as air pollutant variables’ vertical profiles including radiosonde balloons [7], tall towers [8], aircraft [9], small Unmanned Aerial Systems (sUASs, UASs, drones, unmanned aerial vehicles (UAVs)), and rocket-based sounding systems. The most common method of meteorological sounding is radiosondes which are utilized by many national weather forecasting offices as well as airports to gather data that is fed into numerical weather models [10,11,12]. There are a number of commercial options for radiosondes including Vaisaila (Vaisaila Corporation, Helsinki, Finland), iMET (International Met Systems, Grand Rapids, MI, USA), and GRAW (GRAW Radiosondes, Germany) [13]. Radiosonde payloads typically weigh 120 g and are lifted by a helium balloon to altitudes of at least 6000 m (20,000 feet) depending on the size of the balloon and amount of helium added.

Meteorological sounding by rockets (rocketsondes) has been utilized in the past for lower and upper stratospheric atmospheric monitoring [14,15,16,17]. Most current rocket sounding relates to the probing of the upper atmosphere [16,18,19]. Rockets provide a simple way to access higher portions of the atmosphere. Considering flight restrictions and differing rules for rocketry, rocket platforms are a possible area of interest in expanding lower atmosphere sounding technology. While conventional radiosondes use helium balloons for lift, rockets use conventional propellants. Commercial options for rocketsondes for the lower atmosphere appear to no longer be available, though they appear to have been available in the past. Within the United States of America, model rockets (Class 1) are classified as those that use no more than 125 g of slow burning propellant, are made of paper, wood, or breakable plastic, contain no substantial metal parts, and weigh no more than 1500 g completed. Class 1 model rockets require no notification of air traffic control and no height limit (other than that the rocket not become orbital) in the rules, but are limited by the logistical recovery area and other safety concerns specific to the launch [20].

The use of RC aircraft has long been suggested as a way to perform meteorological measurements, dating back to the 1970s [21]. More recently, UASs have been explored and evaluated as viable platforms for lower atmospheric measurements [22] including the use of UASs for systematic meteorological sounding [23]. Several sUAS platforms for meteorological sounding have been reported more recently in the literature [24,25,26,27,28,29,30,31,32,33,34,35]. Likely the most developed of these is the CopterSonde [27] which has a highly integrated measurement system with custom firmware and telemetry for reporting data back to the ground station in real time, as well as integrated software that keeps the sUAS pointed into the wind and reports wind speed and direction based on the inertial measurement unit feedback with an aspirated, shielded set of sensors. It has recently been made available as a commercial system (https://www.intermetsystems.com/products/coptersonde/, accessed on 15 December 2023). Other UAS systems have been developed and tested for measuring a variety of other species such as volatile organic carbon compounds [36,37,38,39,40,41], nitrogen and sulfur oxides [41,42], particulate matter [41,43,44,45,46,47,48], and ozone [46,49,50]. These systems have varied from lower-end instrumentation to high-end research grade instrumentation. Previous works have utilized the Arduino platform for meteorological measurements on sUASs [30,51]. These platforms include both rotary-wing (helicopters and multi-rotors) and fixed-wing aircraft. For sampling, multi-rotors have the advantage in small flight footprints and the ability to ascend and descend easily, along with more simple, easy to use control systems. Fixed-wing UASs typically have longer flight times and distances, sometimes up to or longer than 2 h, which allows for easier horizontal spatial sampling, as well as a variety of fuel types that allow for even longer flight times [44]. Another recent report, for example, detailed a fixed-wing sUAS capable of profiling the atmosphere to a 10 km altitude with a battery powered aircraft [24]. There are also commercial add-on systems for meteorology such as the iMET XQ2 which integrates temperature and relative humidity measurements with pressure and geolocation (GPS) data in an integrated package [52,53,54,55]. This package provides a bead thermistor, and calibrated temperature and relative humidity measurements in a package that is a standalone system.

Within the United States regulatory framework, these systems are referred to as sUASs, indicating the small size, and the integrated airframe and communication and control systems. sUASs have become a common aerial platform for measurements due to their ease of use, portability, and flexibility. sUASs allow the flexibility to sample the immediate boundary layer that is more difficult for some platforms such as manned aircraft to measure and require less infrastructure than a fixed tower. The small size and power limitations do have drawbacks for the platform’s ability to utilize the full suite of chemical analysis that is available for airborne and fixed location systems, where the total airframe and payload per Federal Aviation Administration (FAA) classification for sUASs is less than 55 pounds (24.9 kg). These sUASs are limited to flights in unregulated (Class G) airspace less than 120 m (400 feet) altitude above ground level or above any fixed object (building or tower). These rules are intended to keep unmanned and manned aircraft in separate portions of the airspace to limit hazards to manned aircraft. Additionally, regulations limit flights over people, beyond visual line of sight, over moving motor vehicles, and other pertinent situations. Flights for research purposes fall under the commercial or governmental portions of the regulations, which in general are specified in section 14 of the Code of Federal Regulations (CFR), part 107. In recent years, sUASs operated under Part 107 or operated for recreational purposes but with a mass greater than 250 g must also be equipped with some form of remote identification broadcast module approved by the FAA (RID). Waivers may be submitted to waive the requirements of various parts of the rules, if the operator can prove that the operation may be performed safely (https://www.faa.gov/uas, accessed on 15 December 2023). Flights in regulated airspace can be performed with air traffic control authorization to specific altitudes through the Low Altitude Authorization and Notification Capability (LAANC), or with a certificate of authorization (COA) through the FAA. Other countries have or are adopting similar rules and regulations for their territories.

This work details the testing of a pair of inexpensive temperature and humidity sensors on a sUAS platform for deployment on a model rocket for atmospheric sounding, and aerosol measurements as well as testing of a model-rocket-based Arduino meteorology sensor. Arduino is an open development platform utilizing small single-board computers and various sensor modules that represents an inexpensive and flexible platform for development for researchers and educators without large budgets. This work explores the performance of these sensors relative to a meteorological sensor package under a variety of conditions and the deployment of these sensors on a rocket payload platform.

## 2. Materials and Methods

### 2.1. Prototype sUAS Platform, Sensors, and Flight Planning

**Figure 1:**
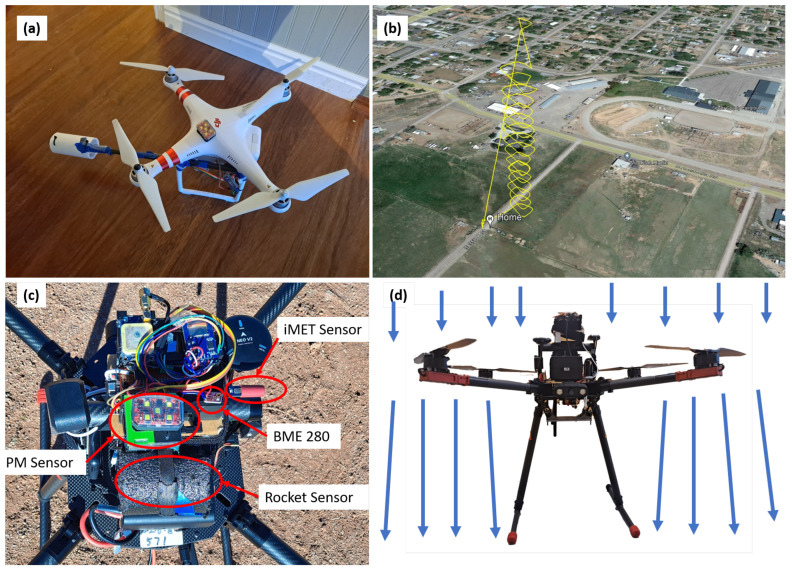
(**a**) DJI Phantom 3 Standard quadcopter with attached Arduino sensor. (**b**) Phantom 3 Standard flight path and altitude profile. (**c**) Arris M900 Quadcopter with attached meteorology sensors. (**d**) Side view showing the typical air movement around the drone during ascent.

Early testing of Arduino sensors (Arduino, Somerville, MA, USA) for meteorological profiling were carried out on a modified DJI Phantom 3 Standard (P3S) UAS (SZ DJI Technology Company Ltd., Shenzhen, China) operated using the Litchi 3rd party control application (VC Technology Ltd., London, UK) for waypoint operation. Temperature, pressure, and relative humidity were measured by a Bosch BME280 sensor (pressure, ±1.7 hPa, 0.18 hPa resolution, temperature: ±0.5 °C (0–65 °C), ±1.25 °C (−20–0 °C), ±1.5 °C (−40–−20 °C), output resolution: 0.01 °C, humidity: 0.008 % RH resolution, 0–100% RH range, ± 3% RH (20–80% RH)). An Arduino Nano board with attached microSD card reader logged the data from each flight. The Arduino assembly was wired directly to the UAS battery with a battery eliminator circuit reducing the voltage to 5 V. The BME280 sensor was located on a boom extending out from the front of the UAS with a foam board cover to shield the sensor from direct sunlight and only allow airflow across the sensor as the UAS moved forward (see Figure 1a). The UAS was then operated by flying a forward circuit at 10 m intervals to the maximum flight height (see Figure 1b). For most flights the maximum flight height was 140 m AGL (120 m above nearby fixed objects or buildings). One test flight was executed near a radio broadcast tower with a height of 87 m (287 feet) so the maximum altitude was 207 m, staying under the overlying Class E controlled airspace beginning at 213 m (700 feet).

### 2.2. sUAS Platforms and Flight Planning

**Figure 2:**
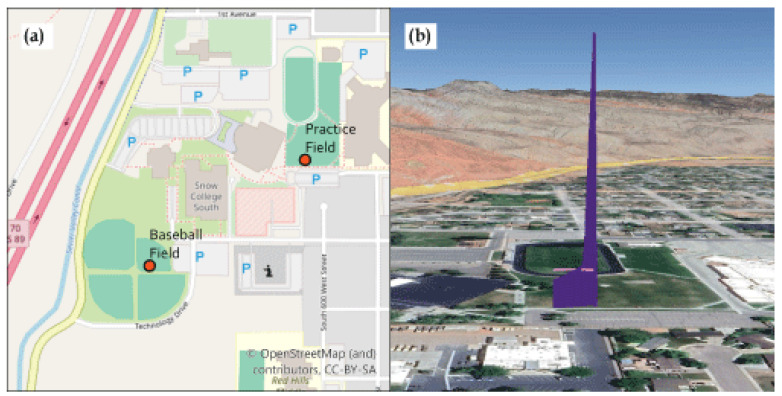
(**a**) Map showing the locations of the test flights from 13 to 14 October 2023. (**b**) Side view showing the height of the flight path.

The meteorological and air quality sensors were mounted on an Arris M900 quadcopter (Arris Hobby, Chengdu, Sichuan, China, 700 mm × 700 mm × 600 mm frame, 6S 22,000 mAh battery, CUAV Pixhack V5 Plus flight controller (CUAV, Guangzhou, China), 220 kV motors, 56 cm (22 inch) propellers, and a maximum takeoff weight of 10 kg). All sensors sampled from the top, center of the sUAS (see Figure 1). The flight route was planned in the built-in app (a derivative of QGroundControl (DroneCode Project, Inc., San Francisco, CA, USA)) with a manual take off, then proceeding to 10 m altitude, followed by a straight vertical flight from 10 m to 140 m. The flights were executed in one of two locations: near a tall building or in a set of baseball diamonds with stadium lighting poles in Richfield, Utah, USA (38.76636 N, 112.09778 W) as shown in Figure 2. The neighboring building or the floodlight poles were used to increased the allowed flight ceiling from 120 m to 140 m per FAA regulations. Flights for this work were carried out 13–14 October 2023. Night flights used strobe lighting visible for at least 3 statute miles.

### 2.3. Meteorology Sensor

The iMet-XQ2 UAV Sensor (InterMet Sensors, Grand Rapids, MI, USA) was used as a reference measurement due to its self-contained packaging and sensors. The XQ2 contains a piezoresistive pressure sensor with a 10 ms response time, a resolution of 0.01 hPa, and an accuracy of ± 1.5 hPa. The relative humidity was measured by a capacitive sensor with resolution of 0.1% RH, an accuracy of ± 5% RH, and response time varying from 0.6 s at 25 °C to 10.9 s at −10 °C. The temperature sensor is a bead thermistor with a 1 s response time at 5 m/s flow, resolution of 0.01 °C, and an accuracy of ±0.3 °C. The time was read from the included global positioning system (GPS). The GPS was a UBlox (Thalwil, Switzerland) CAM-M8 with a vertical accuracy of 12 m and a 1 s response time. The sensor probe was positioned on top of the UAS in the clear airflow. No shielding or aspiration [30] was utilized as the measurements were being used in comparison to the cheaper sensors.

### 2.4. Rocket Arduino Sensor Package

A second Arduino-based sensor built for use on a model rocket was flow on the top of the UAS using a similar board, Bosch BME280 sensor (Bosch Sensortec, Reutlingen, Germany), GPS, and an additional thermocouple module to provide a faster time response temperature measurement. In the first set of flights, this sensor package did not have the RH sensor exposed to the outside air directly but was situated in the second set of launches so that the BME 280 sensor was exposed to the outside air. The thermocouple sensor was a type K thermocouple attached to a MAX31865 amplifier board (Adafruit, New York, NY, USA, resolution 0.007 °C, readout resolution 0.01 °C, accuracy ±1.5 °C). Data were written at 2 Hz to a microSD card. The thermocouple sensor output was adjusted with a scalar offset relative to the ground-level temperature for the bead thermister from the iMET sensor for each flight to account for any changes in the cold-junction temperature of the thermocouple.

### 2.5. Arduino-Based Meteorology Sensor

An Arduino (Arduino LLC) sensor was attached above the iMET sensor controlled by a Mega 2560 microprocessor board (AITRIP Mega 2560, CH340G/ATMEGA2560-16AU). The processing board and sensors were housed in a 3D-printed housing that attached to the handle on the top of the UAS. The sensor was powered from a separate battery pack (5V). Custom code was used to read and log measurements from external sensors to a microSD memory card. Pressure, temperature, and relative humidity were measured by a Bosch BME280 sensor as described in the previous set up. The module utilized a GPS positioning antenna to read altitude, latitude, and longitude, as well as the time and date. The temperature and relative humidity response of the BME280 sensor was calibrated relative to the iMET sensor to account for any offsets.

The sensor also integrated a Sensirion SPS30 particulate matter (PM) sensor (Sensirion AG, Stafa, Switzerland). The module reports at a 1 s interval values of PM1.0, PM2.5, PM4.0, and PM10, as well as number concentration bins of 0.3–0.5 μm, 0.3–1.0 μm, 0.3–2.5 μm, 0.3–4.0 μm, and 0.3–10.0 μm. Unfortunately, it has been found that the PM4 and PM10 data are extrapolated from the the distribution of all of the particles [56,57,58]. The larger size bins are estimated because the number of particles in those bins is small and would lead to large variation in the reported values since only a few particles had been counted based on the volume of air sampled. The uncertainty for the lower size bins (up to 2.5 μm) is ±10%, ±100 cm^−3^, or ±10 μg/m^3^, whichever is larger, depending on the magnitude of the measurement.

### 2.6. Data Analysis

**Figure 3:**
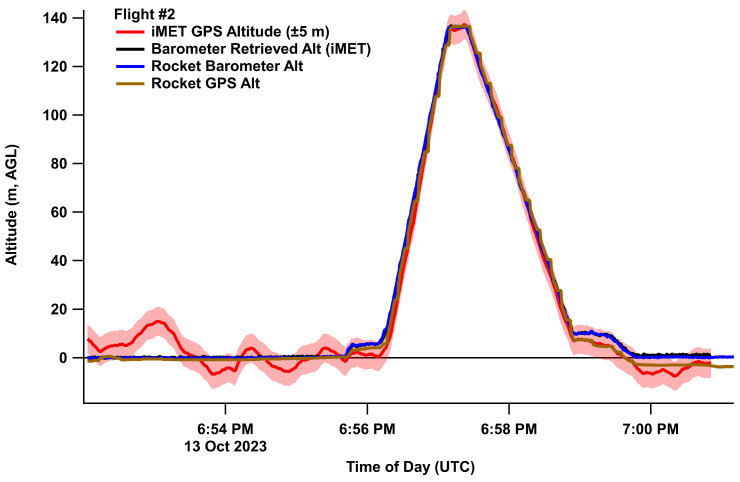
Retrieved altitude from the GPS and barometric sensors. Uncertainty in the GPS signal is given as ±6 m. The barometric sensor signals lead to better precision and less noise than the GPS.

For each separate instrument, altitude above ground level (AGL) was retrieved from the measurement of pressure using the hypsometric formula [59]:

(1) $$AGL=Alt_{0}+\frac{T_{b}}{L_{b}}\left[{\frac{P_{0}}{P_{i}}}^{\frac{-RL_{b}}{g_{0}M}}-1\right]$$

where the altitude (AGL, above ground level) is in meters, P_0_ is the initial pressure at the ground level, P_i_ is the current pressure at a given height, R is the universal gas constant (8.314 N m mol^−1^ K^−1^), g_0_ is the gravitational constant (9.80665 m s^−2^), M is the molar mass of air (0.0289644 kg m^−3^), T_b_ is set to average temperature of the profiled column in Kelvin, and Alt_0_ is given as zero to scale to the height AGL. This assumes a constant lapse rate (L_b_, −0.0065 K m^−1^). The retrieved altitude from the pressure has significantly lower noise relative to the altitude retrieved by the GPS receivers (Figure 3).

For each flight, the data were filtered to leave only the ascending data starting at 10 m above the starting point. The UAS was programmed to leave the takeoff point and fly to the first waypoint in the field which was at an altitude of 10 m, then to ascend vertically to 140 m, wait 10 s at that point, and then descend back to 10 m. The remote pilot in command (RPIC) then took manual control to land the aircraft. All flights were performed under 14 CFR Part 107 rules and regulations. The ascending data were taken at the start of the ascent after reaching the first waypoint until the UAS reached maximum altitude. This ensured that fresh, unperturbed air had a better chance of interacting with the sensors. There have been a number of studies investigating sensor location on multi-rotor vehicles, with the simplest being to locate the sensors on the top of the UAS and only use data from when the UAS gains altitude [30,35].

Data were then averaged by height into 10 m vertical bins for viewing. Data processing and analysis for this work were performed in IGOR Pro (Wavemetrics, Lake Oswego, OR, USA).

### 2.7. Rocket Flights

**Figure 4:**
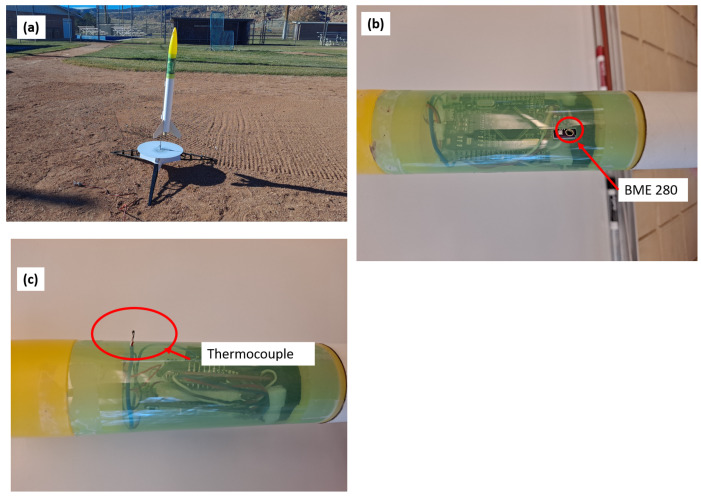
Configuration and set up of the rocket as shown by (**a**) the rocket on the launch pad ready for launch, (**b**) BME 280 sensor exposed through payload housing, (**c**) thermocouple sensor protruding from rocket payload section exposed to outside air during flight.

The Rocket sensor was then deployed on an Estes (Estes Rockets, Penrose, CO, USA) GreenEggs model rocket which includes a payload bay. A small hole was made in the plastic payload bay to allow the thermocouple to protrude and for the pressure to equalize between the outside and the interior pressure (altitude) sensor. The rocket was then launched using a D12-3 rocket engine (Estes, 12 newton impulse, 3 s delay for parachute deployment). A later flight (29 January 2024) utilized an E-16-5 rocket engine (Estes) to test possible higher heights for the rocket with the BME-280 sensor also exposed to the outside air to be able to measure RH. The sensor was powered using two 30 mAh 1S lithium polymer batteries connected in series to generate a voltage of greater than 7.5 for the Arduino board to power on through the voltage in (VIN) pin. Meteorology data were retrieved from the parachute-enabled descent of the rocket. Rocket launches paired with UAS flights were performed on 12 (1:35 PM) and 15 December (8:37, 8:42, 8:47, and 8:55 AM), 2023, and 29 January 2024 (3:00 PM). Sensor integration in the rocket payload bay is shown in Figure 4.

## 3. Results

### 3.1. Prototype UAS System Results

**Figure 5:**
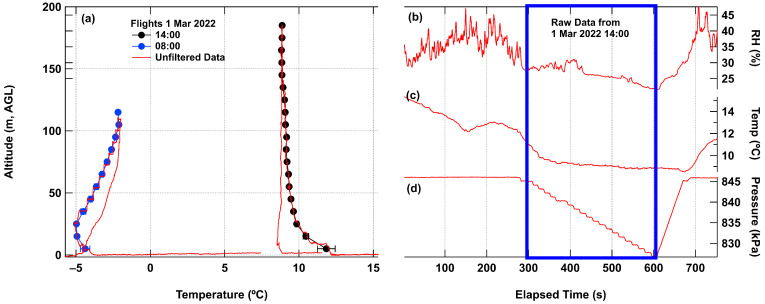
Retrieved data from prototype flights including (**a**) binned temperature data including raw data showing both ascending and descending flight, (**b**) relative humidity, (**c**) temperature, and (**d**) pressure. The blue box highlights the section of the flight where the UAS is ascending from 1 March 2022.

While many flights were performed with the P3S prototype system, two flights are shown here from 1 March 2022 as examples of results and to highlight issues relative to this version of the platform and sensor (see Figure 5). For an inverted temperature profile (AM flight), the descending flight diverged from the ascending flight with warmer temperatures, while on the afternoon flight with a more standard lapse rate (PM flight) the descending flight temperature was cooler than the ascending flight.

### 3.2. UAS Test Flight Results

Flights were performed near the hour as given in Table 1. Two different locations adjacent to the school were used for flights depending on the availability of the fields for operations. Flights were performed throughout the day and night time as listed in Table 1.

**Figure 6:**
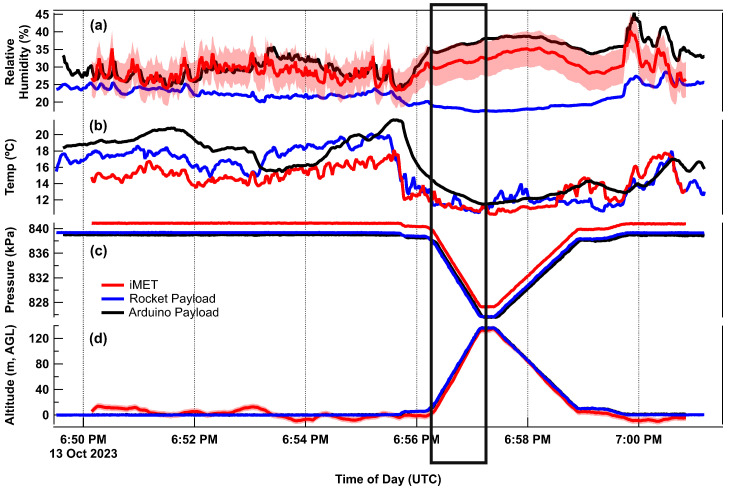
Retrieved data from Flight #2 including (**a**) altitude, (**b**) pressure, (**c**) temperature, and (**d**) relative humidity. The black box highlights the section of the flight where the UAS is ascending.

Data from all five flights were recorded and processed (see Table 1). Corrections to the temperature and relative humidity were made to adjust values relative to the iMET sensor. Individual flight data show the response of all of the sensors to changes in the temperature either on the ground or during the vertical flight as seen in Figure 6.

**Figure 7:**
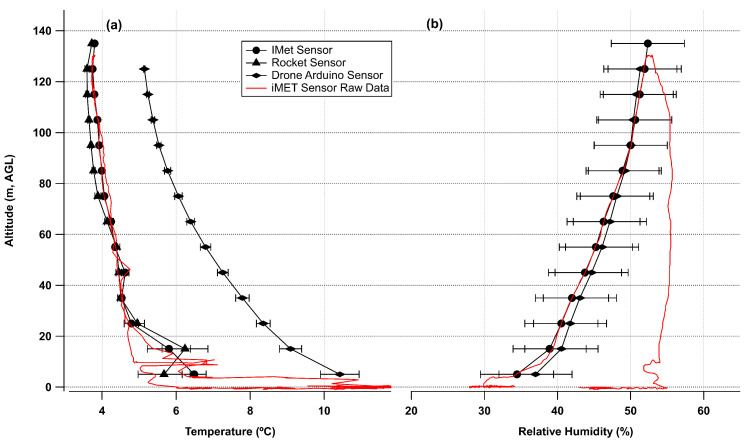
Binned data from Flight #2 showing (**a**) temperature and (**b**) relative humidity. Error bars for temperature are variability from the average of the altitude bin. Error bars for RH show the absolute accuracy of the RH measurements ±5% RH.

Data from each flight were then binned into vertical bins of 10 m starting at 0–10 m and continuing to the maximum altitude reached. The first bin usually contained a few points right as the UAS started to ascend. Figure 7 shows the binned data from Flight #2 for both temperature (iMET and Rocket) and RH (iMET and Arduino). Raw data from the iMET sensor are shown to illustrate the variability and difference between the ascending and descending portion of the flight. In this flight the BME280 sensor showed a significant lag in temperature response relative to the other two sensors.

**Figure 8:**
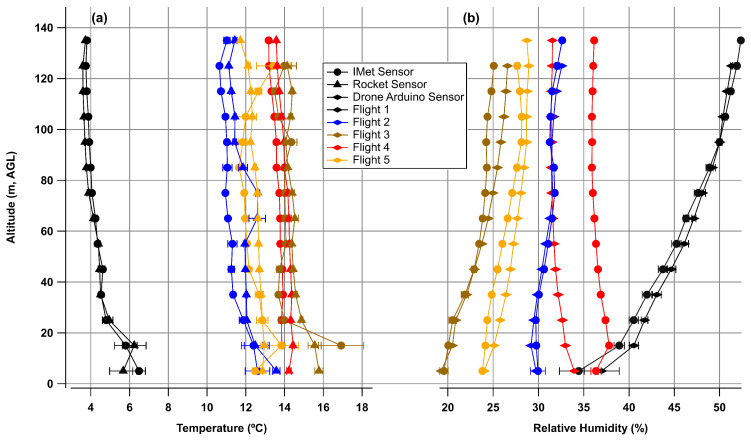
Binned data from all flights for (**a**) temperature and (**b**) relative humidity. Error bars for temperature and relative humidity are variability from the average of each altitude bin.

The five flights over the course of 24 hours show similar trends in the comparison of the three sensor packages as shown in Figure 8.

### 3.3. Rocket Test Flight Results

**Figure 9:**
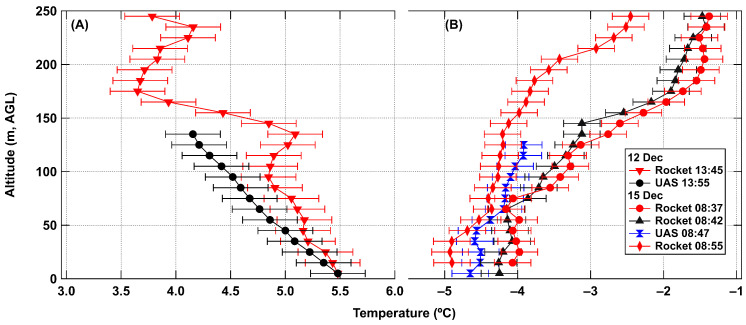
Binned data from rocket tests for (**A**) afternoon flight with normal lapse rate and (**B**) a set of morning flights showing an inverted temperature profile. Error bars for temperature are ±0.25 °C.

The rocket flew to a height of 245–247 m AGL during the test flights and descended by parachute back to the ground. Data from the descent were processed and showed a similar trend to data from the iMET sensor for both standard and inverted temperature profiles as seen in Figure 9. During the second launch on 15 December, the parachute failed to deploy but the ejection charge still separated the two portions of the rocket. This provided a faster descent but still at a fairly controlled speed.

**Figure 10:**
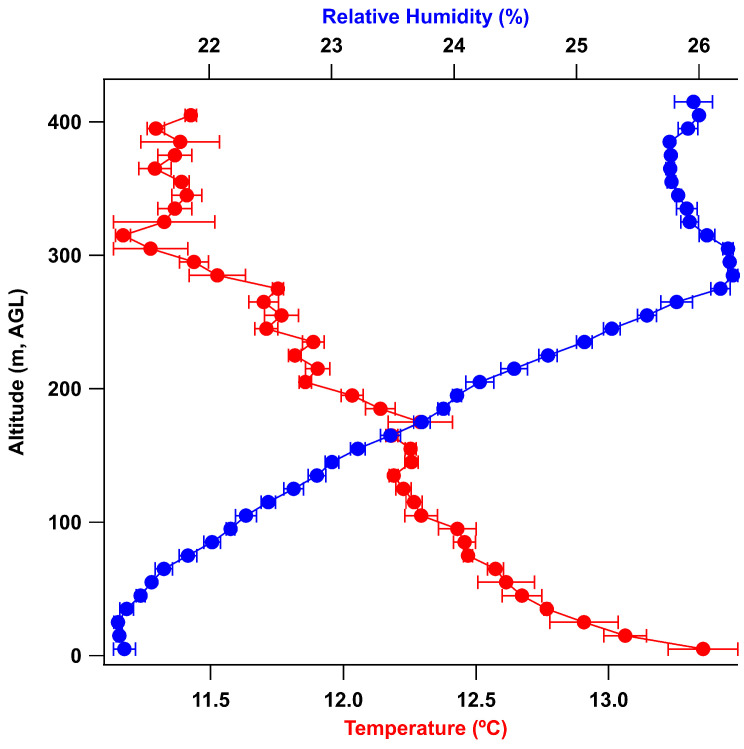
Binned data from rocket tests for 29 January 2024, showing the temperature and relative humidity, and sampled height of 400 m.

A second set of test flights was performed on 29 January 2024 using an E rocket engine. During these flights the rocket achieved an apogee of 403 m with successful parachute deployment and sampled the temperature and relative humidity on the descent as shown in Figure 10.

### 3.4. Aerosol Measurements

**Figure 11:**
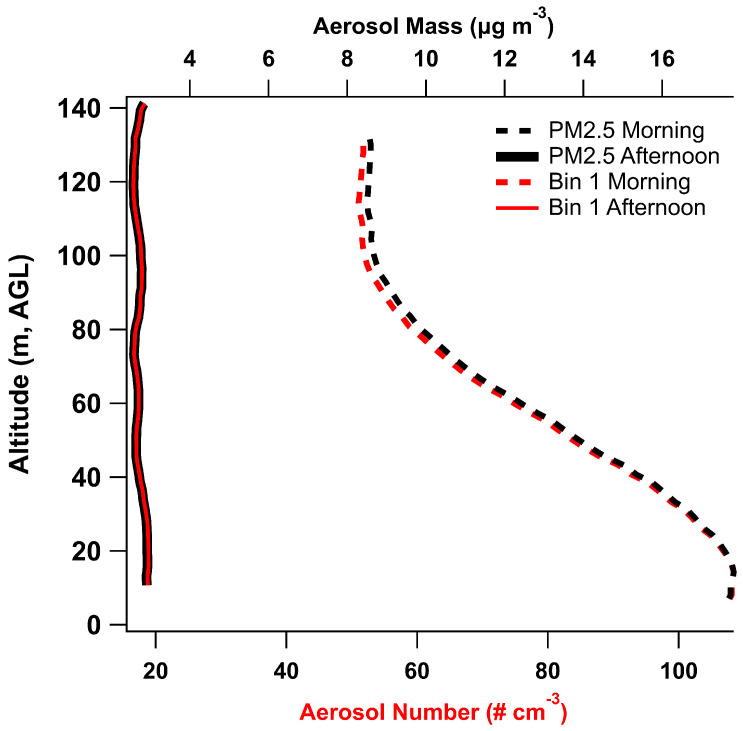
Particulate data from 12 December 2023 showing morning and afternoon flights.

During the December UAS platform tests, the SPS30 sensor retrieved aerosol size and calculated mass concentration with respect to height. Data from 12 December 2023 flights that accompanied the rocket flights for the same day are shown in Figure 11. The performance of the sensor data is evaluated using the PM2.5 mass concentration and the smallest size bin number concentration for the inversion (AM) data and the boundary layer mixing (PM) data as shown in Figure 11.

## 4. Discussion

### 4.1. Prototype Platform

The prototype P3S Arduino platform performed well for the set up but had several disadvantages. The system did not include a GPS or other timing device to provide timestamps for the data; this was corrected in the next version by adding a GPS unit, but could also be corrected using a real time clock (RTC) or a different Arduino board that includes an RTC (as in [51]). The complicated flight plans allowed for continually sampled air to move over the sensor, but occupy more time and planning. Figure 4a clearly shows the effect of the quadcopter mixing the air in the vertical column as the descending flights are biased toward the temperature at the highest point in the flight (warmer descending temperature for an inverted profile and cooler descending temperature for a normal profile). There appears to be a range of temperatures at each flight level as the UAS completes the loop at each elevation. This type of flight pattern adds time, which may limit flight times if the UAS is allowed to fly to much higher. This sensor position also does not lend itself to mounting of larger sensors which would cause greater instability in the flight of the UAS. The preliminary data from this prototype led to the sampling arrangement for the Arris M900 UAS with top-mounted sensors, data collection only on the ascending flight, and a faster ascent.

### 4.2. Meteorology Sensor Comparison

**Figure 12:**
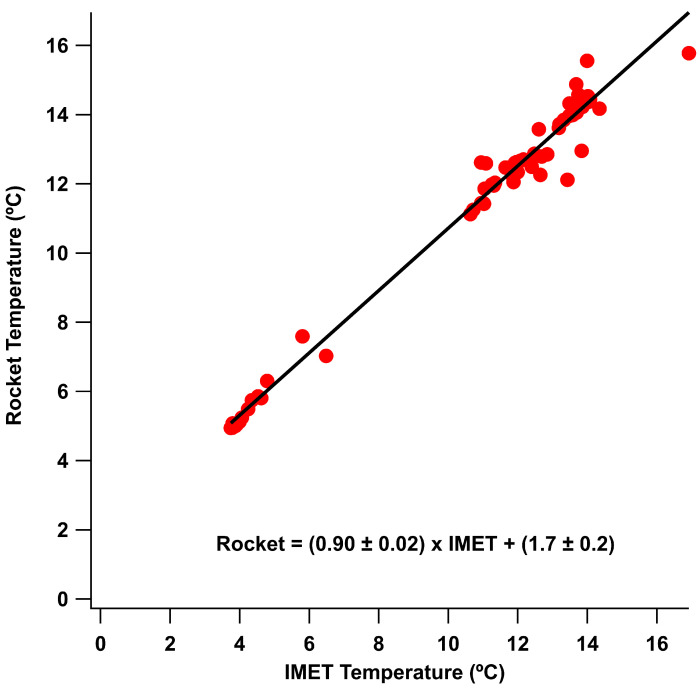
Correlation of temperature data from all five sUAS flights between the IMET and rocket sensor with a slope of 0.90, an offset of 1.7, and an R^2^ of 0.98.

For comparison to the iMET meteorology sensor, the Arduino Bosch BME280 sensor performed well for measurements of atmospheric pressure and relative humidity. The temperature measurement of the BME280 while following the trend of the bead thermister took much longer to equilibrate to the current temperature and also appeared to receive warming from solar radiation. The thermocouple sensor on the Arduino was more responsive to changes in time and matched well with the bead thermister on the iMET sensor. The variation of the BME280 temperature sensor had more to do with the time the UAS had been outside prior to flight than any other factor as the lag in response was not as pronounced in all flights. The highest variability flight was where the UAS had been brought immediately outside, while on the other flights the UAS had been outside for more than 20 min prior to flight. This is difficult to replicate in cold weather where lithium polymer batteries should not be operated from a cold start which will dramatically impact the lifespan of the battery. This is one limitation that can be overcome by keeping the batteries above 20 °C but allowing the rest of the UAS to be at ambient temperature. The thermocouple sensor provided a faster response in flight relative to the BME280 sensor. The BME280 sensor is mounted on a small metal printed circuit board (PCB) plate which means the temperature of the larger PCB has to change for the sensor to register the change. The IMET and thermocouple sensors compared linearly as seen in Figure 12, with variation likely caused by drift in the cold-junction temperature of the thermocouple [27] or issues with radiation shielding [30]. While previous authors used a variety of methods to shield the temperature sensors from short- and long-wave radiation, in this work, where the comparison and feasibility of the cheaper sensors were tested, no radiation shield was utilized. For future measurements from the UAS platform, shielding and aspiration across the sensor by moving the sensor under the propeller for the vertical ascending flights will be necessary as outlined in Inoue and Sato [30]. Shielding as outlined in Inoue and Sato [30] requires active aspiration from the propellers as the vertical flight speed does not generate enough airflow to aspirate the sensors inside the radiation shield.

### 4.3. Rocket Sensor System

**Figure 13:**
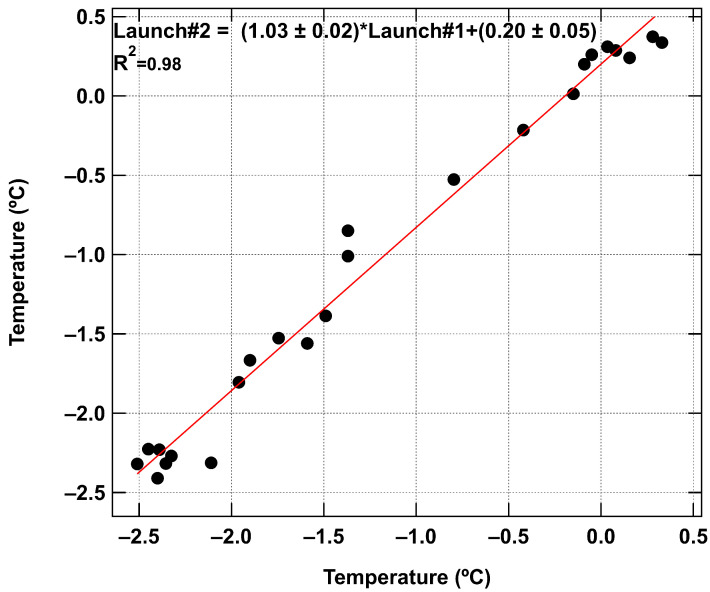
Correlation of temperature data from launches 1 and 2 from 15 December shown with a slope of 1.02, an offset of 0.2, and an R^2^ of 0.98.

The descent of the rocket, both with and without parachute deployment, was able to capture a detailed vertical temperature profile for both standard and inverted temperature gradients. The two flights on 15 December with the shortest time between (5 min) also showed very good agreement, showing good reproducibility between flights as seen in the correlation in Figure 13.

### 4.4. Particulate Measurements

The SPS30 sensor attached to the Arduino sensor located on the top of the Arris M900 UAS was able to capture the trapped aerosol in the lower boundary layer inversion as observed on 12 December 2023 (Figure 10). The following afternoon flight showed that mixing had occurred with an evenly distributed aerosol concentration with height in the afternoon with the boundary layer height higher than the UAS flew. In particular, this shows the utility of even lower end sensors in providing information about the aerosol vertical distribution, especially when inversions are observed.

## 5. Conclusions

The rocket-based system provides a simple method to acquire lower atmosphere temperature sounding without the use of helium and with a reusable sensor package. Other larger rockets that still fall within the Class 1 model rocket designation could be used to probe to higher altitudes on a repeatable basis. The UAS platform can also integrate larger sensors for detecting particles or trace gases when paired with the baseline meteorological measurements as demonstrated with the small Sensiron SPS30 particle sensor. UAS systems can fly to higher heights through a waiver process but it has proven to be time consuming to get the waiver approved. The UAS systems if approved for higher flights provide vertical profile information without the need for as large an area for payload recovery and without a per-flight cost of rocket motors. The Arduino connected cheap sensors described here allowed for the measurement of meteorological variables from an open source sensor platform with a similar response to the iMET sensor and are viable options for platform development for meteorology and air quality measurements.

Future work includes the retrieval of wind speed and direction from the descent of the rocket sensor from the GPS data, the application of both rocket and sUAS platforms to smaller budget air sampling campaigns to understand the boundary, and the use of the sensors for meteorology and field sampling classes to understand the evolution of the planetary boundary layer. Further development of the rocket platform and sensor may also include incorporating a different processor other than Arduino (Raspberry Pi or Micropython for example) or combining the elements on a single printed circuit board system to yield more dependable GPS data and greater ease of operation.

## Figures and Tables

**Table 1:** Flights and flight times for UAS tests 13–14 October 2023.

Flight Number	Time of Day (MST)	Location
1	9:00	Practice Field
2	12:00	Baseball Field
3	13:45	Baseball Field
4	19:45	Baseball Field
5	12:00 (14-October)	Baseball Field

## Data Availability

Data for this project can be found in the related Supplementary Materials.

## Supplementary Materials

The following supporting information can be downloaded at: https://www.mdpi.com/article/10.3390/s24061768/s1, Graph Data: igor text files that correspond to the data found in all of the figures in the paper.

## Abbreviations

The following abbreviations are used in this manuscript:

UAS	Unmanned Aerial System
sUAS	small Unmanned Aerial System
RH	relative humidity
NASA	National Aeronautics and Space Administration
RPIC	remote pilot in command
FAA	Federal Aviation Administration
CFR	Code of Federal Regulation
P3S	Phantom 3 Standard (DJI sUAS)
